# Clinical and Angiographic Characteristics of Elderly and Very Elderly Patients With Coronary Artery Disease

**DOI:** 10.7759/cureus.113459

**Published:** 2026-07-27

**Authors:** Suraj Khanal, Arkaru Pradeep, Basant Kumar, Ravinish Kumar

**Affiliations:** 1 Cardiology, Postgraduate Institute of Medical Education and Research, Chandigarh, IND; 2 Internal Medicine, Postgraduate Institute of Medical Education and Research, Chandigarh, IND

**Keywords:** cardiovascular risk factors, coronary angiography, elderly, multivessel coronary disease, percutaneous coronary intervention, very elderly

## Abstract

Background: Coronary artery disease (CAD) is a major cause of morbidity and mortality worldwide, with an increasing burden among older adults. Very elderly patients (≥80 years) often present with atypical symptoms and more extensive coronary involvement yet remain underrepresented in clinical studies. The aim of this prospective observational study was to compare the demographic, clinical, echocardiographic, and coronary angiographic characteristics of elderly (60-79 years) and very elderly (≥80 years) patients with CAD, with the objective of describing age-related clinical profiles that may assist in the evaluation of this increasingly important patient population.

Methods: This prospective, observational, single-center study enrolled consecutive patients aged ≥60 years presenting with CAD, including unstable angina, non-ST-elevation myocardial infarction (NSTEMI), ST-elevation myocardial infarction (STEMI), and chronic stable angina. Clinical characteristics, cardiovascular risk factors, echocardiographic findings, and coronary angiographic data were recorded and compared between elderly (60-79 years) and very elderly (≥80 years) groups. CAD was classified as obstructive (≥50% stenosis) or non-obstructive (<50% stenosis).

Results: A total of 57 patients were included, comprising 30 patients aged 60-79 years and 27 patients aged ≥80 years. Hypertension (43, 75.4%), diabetes mellitus (26, 45.6%), and dyslipidemia (25, 44.0%) were the most prevalent cardiovascular risk factors. Dyspnea (36, 63.2%) and angina (29, 50.9%) were the most common presenting symptoms. Echocardiographic findings and left ventricular ejection fraction were comparable between groups. Coronary angiography demonstrated single-vessel disease predominantly in patients aged 60-79 years (19, 63.3%), whereas double-vessel disease was more common among very elderly patients (11, 40.7%). The left anterior descending artery was the most frequently involved vessel in both groups. Percutaneous coronary intervention was the predominant management strategy in both elderly (24, 80.0%) and very elderly patients (19, 70.4%).

Conclusion: Our study demonstrates that very elderly CAD patients exhibit more extensive coronary involvement, particularly double-vessel disease, despite similar left ventricular function.

## Introduction

Coronary artery disease (CAD) remains the leading cause of morbidity and mortality worldwide and represents a major public health challenge in both developed and developing countries, including India. CAD is a multifactorial disorder resulting from complex interactions between non-modifiable risk factors, including advancing age, sex, and genetic predisposition, and modifiable risk factors such as hypertension, diabetes mellitus, dyslipidemia, cigarette smoking, obesity, and sedentary lifestyle [1,2]. These factors contribute to endothelial dysfunction, chronic vascular inflammation, and progressive atherosclerotic plaque formation, ultimately leading to myocardial ischemia and acute coronary syndromes. Diabetes mellitus, in particular, is among the strongest independent predictors of CAD and is associated with accelerated atherosclerosis, diffuse coronary involvement, endothelial dysfunction, and poorer cardiovascular outcomes. Hypertension further promotes vascular remodeling and plaque progression, while dyslipidemia and smoking accelerate plaque instability and thrombotic complications. The cumulative burden of these cardiovascular risk factors becomes increasingly important with advancing age, contributing to both the incidence and severity of CAD [3].

Inflammation plays a central role in plaque instability, with markers like high-sensitivity CRP linked to rupture risk. Aging is a major risk factor, and with increased life expectancy, CAD incidence is rising among the elderly [4,5]. Despite substantial advances in prevention and revascularization strategies, very elderly patients (≥80 years) remain underrepresented in major cardiovascular studies. Compared with younger elderly individuals, octogenarians frequently present with atypical symptoms, delayed diagnosis, multiple comorbidities, and more extensive CAD, making clinical decision-making particularly challenging [6]. A better understanding of the demographic profile, cardiovascular risk factors, clinical presentation, and angiographic characteristics of elderly and very elderly patients may facilitate earlier diagnosis, individualized risk stratification, and more appropriate therapeutic strategies. Therefore, the aim of this prospective observational study was to compare the demographic, clinical, echocardiographic, and coronary angiographic characteristics of elderly (60-79 years) and very elderly (≥80 years) patients with CAD, with the objective of describing age-related clinical profiles that may assist in the evaluation of this increasingly important patient population.

## Materials and methods

Study design and population

**Figure 1:**
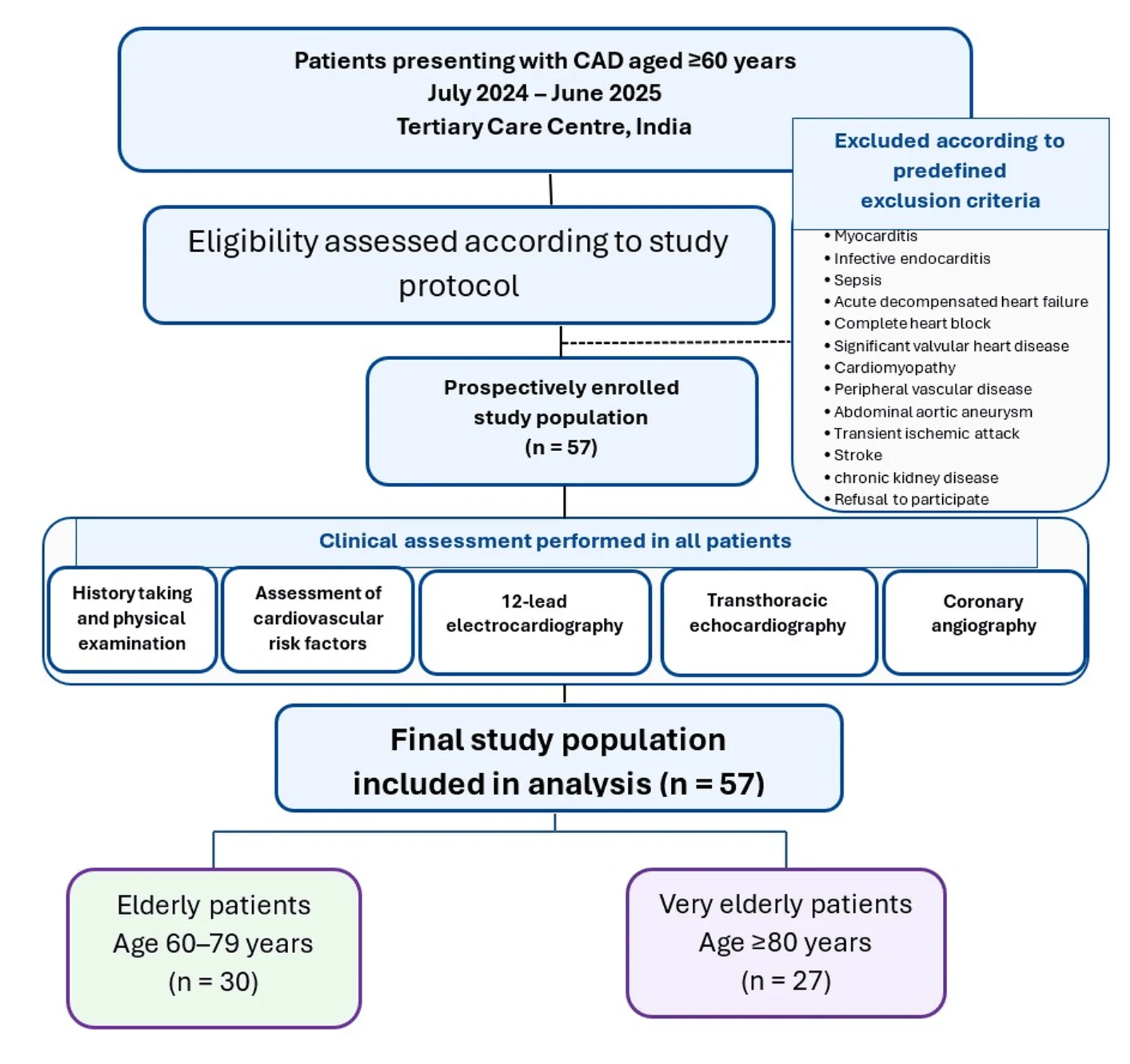
Study flow diagram illustrating patient selection, exclusion, and final study population. CAD: coronary artery disease.

This was a single-center, prospective, observational study conducted at a tertiary care center in India from July 2024 to June 2025. Adults (≥60 years) presenting with CAD, including unstable angina, non-ST-elevation myocardial infarction (NSTEMI), ST-elevation myocardial infarction (STEMI), and chronic stable angina, who met standard indications were prospectively enrolled. Key exclusion criteria were myocarditis, infective endocarditis, sepsis, acute decompensated heart failure, complete heart block, valvular heart disease or cardiomyopathy, or patient refusal. Patients with a past history of peripheral vascular disease (PVD), abdominal aortic aneurysm, transient ischemic attack (TIA), stroke, and chronic kidney disease (CKD) were excluded. The study was approved by the Institutional Ethics Committee of Postgraduate Institute of Medical Education and Research (IEC-INT/2024/MD-1644) on 2 July 2024, and all participants provided informed consent (Figure 1).

Clinical assessment

Baseline data collection included detailed history and physical examination with documentation of demographics and coronary risk factors (hypertension, diabetes mellitus, smoking status, dyslipidemia, and family history of CAD). A 12-lead electrocardiogram (ECG) was obtained in all cases, and transthoracic echocardiography was performed to assess left ventricular (LV) function and to look for regional wall motion abnormalities (RWMAs) suggestive of prior myocardial infarction. Transthoracic echocardiography was performed according to routine institutional clinical practice. RWMAs were recorded based on the corresponding coronary artery territory (left anterior descending, right coronary artery, or left circumflex artery) as documented in the echocardiography reports.

Coronary angiography

All patients underwent invasive coronary angiography during the index hospitalization. Coronary angiograms were performed via a transradial or transfemoral approach and interpreted by two experienced cardiologists blinded to the patients’ clinical details. CAD was defined as the presence of any atherosclerotic narrowing in at least one major epicardial coronary artery or their first-order branches. Obstructive CAD was defined as ≥50% diameter stenosis in any vessel, while non-obstructive CAD comprised plaque causing <50% stenosis. Based on the angiographic findings, patients were categorized into three groups: (1) obstructive CAD: significant stenosis ≥50% in at least one vessel; (2) non-obstructive CAD: presence of plaque <50% without significant stenosis; (3) normal coronaries: no angiographic evidence of CAD. For patients with obstructive CAD, the number of diseased vessels was recorded (single-vessel, double-vessel, or triple-vessel disease).

Sample size calculation

The sample size was calculated using the formula described by Lemeshow [7] based on an expected prevalence of STEMI of 62.04%, assuming a 95% confidence level and an absolute precision of 10%, yielding a required sample size of 91 participants. However, due to recruitment constraints, a total of 57 patients were enrolled during the study period.

Sample size \( n = \frac{\left(Z_{1-\alpha/2}\right)^2 \times p \times (1-p)}{\delta^2} \)

Proportion of subjects with STEMI (most common cause): p = 0.6204 (62.04%)

Precision: δ = 0.10 (10%)

Type I error: α = 0.05 (5%), Z(1−α/2) = 1.96; beta = 20%, and power: 80%

\( n = \frac{(1.96)^2 \times 0.6204 \times (1 - 0.6204)}{(0.10)^2} \)

N = 90.47

Accordingly, the required minimum sample size was rounded up to 91 participants.

Statistical analysis

Data were summarized using descriptive statistics. Continuous variables are presented as mean ± standard deviation (SD), whereas categorical variables are expressed as frequencies and percentages. The present study was primarily intended to describe and compare the demographic, clinical, echocardiographic, and coronary angiographic characteristics of elderly and very elderly patients with CAD. Owing to the exploratory nature of the study and the relatively small sample size, the findings are presented primarily as descriptive observations.

## Results

Patient characteristics

A total of 57 patients (mean age = 75.53 ± 8.43 years) in the study were split into two groups: 30 patients aged 60-79 years (52.6%) and 27 patients aged ≥80 years (47.4%). The cohort had a high prevalence of coronary risk factors: 43 patients (75.4%) had hypertension, 26 patients (45.6%) had type 2 diabetes, 25 (44%) had dyslipidemia, and nine (15.8%) were current or former smokers. Family history of CAD was present in 19 patients (33.3%). Clinically, dyspnea (n = 36, 63.2%) and angina (n = 29, 50.9%) were the most common presenting symptoms, followed by nervousness (n = 27, 47.4%), typical chest pain (n = 18, 31.6%), and palpitations (n = 1, 1.8%) (Table 1). Baseline echocardiography revealed left anterior descending artery (LAD) territory hypokinesia in 23 patients (40.4%), right coronary artery (RCA) territory hypokinesia in 17 patients (29.8%), left circumflex (LCx) territory hypokinesia in two patients (3.5%), and global hypokinesia in six patients (10.5%), while 17 patients (29.8%) had normal findings (Table 2). Ejection fraction (EF) <40% was observed in 30.0% (n = 9) of patients aged 60-79 years and 33.3% (n = 9) of those aged ≥80 years, while an EF of 40-50% was present in 33.3% of both groups (n = 10 in 60-70 years; n = 9 in ≥80 years). Preserved systolic function (EF >50%) was noted in 11 patients (36.7%) and nine patients (33.3%), respectively, indicating no significant intergroup difference in left ventricular systolic performance (Table 3).

**Table 1:** Baseline demographic and clinical characteristics. Data are presented as mean ± SD or n (%).

Variables	N = 57 patients
Mean age, years	75.53 ± 8.43
Hypertension	43 (75.4)
Diabetes mellitus	26 (45.6)
Dyslipidemia	25 (44.0)
Smoking history	9 (15.8)
Family history of coronary artery disease	19 (33.3)
Dyspnea	36 (63.2)
Angina	29 (50.9)
Typical chest pain	18 (31.6)
Nervousness	27 (47.4)
Palpitations	1 (1.8)

**Table 2:** Echocardiography findings. Data are presented as n (%). LAD: left anterior descending artery; RCA: right coronary artery; LCx: left circumflex.

Variables	N = 57 patients
LAD territory hypokinesia	23 (40.4)
RCA territory hypokinesia	17 (29.8)
LCx territory hypokinesia	2 (3.5)
Global hypokinesia	6 (10.5)
Normal echocardiography	17 (29.8)

**Table 3:** Ejection fraction based on age among patients with coronary artery disease. Data are presented as n (%).

Ejection fraction	Total N = 57 patients	60-79 years, n = 30 patients	≥80 years, n = 27 patients
<40%	18 (31.6)	9 (30.0)	9 (33.3)
40-50%	19 (33.3)	10 (33.3)	9 (33.3)
>50%	20 (35.1)	11 (36.7)	9 (33.3)

Incidence and extent of CAD

In patients aged 60-79 years (n = 30), angiography showed plaque in three patients (10.0%), single-vessel disease in 19 patients (63.3%), double-vessel disease in one patient (3.3%), and triple-vessel disease in seven patients (23.3%). Among those aged ≥80 years (n = 27), plaque was observed in five patients (18.5%), single-vessel disease in nine patients (33.3%), double-vessel disease in 11 patients (40.7%), and triple-vessel disease in two patients (7.4%). These findings indicate an age-related shift from single vessel predominance in the younger elderly to double vessel predominance in the very elderly. Revascularization strategies were broadly similar across age groups. In the 60-79 years group, percutaneous coronary intervention (PCI) was performed in 24 (80.0%), coronary artery bypass grafting (CABG) in one (3.3%), and optimal medical therapy in five (16.7%) patients. In the ≥80 years group, PCI was undertaken in 19 patients (70.4%), CABG in one patient (3.7%), and optimal medical therapy in seven patients (25.9%). Overall, 43 (75.4%) patients underwent PCI, two (3.5%) underwent CABG, and 12 (21.1%) underwent optimal medical therapy (Table 4).

**Table 4:** Coronary angiography findings and treatment strategy. Data are presented as n (%). CABG: coronary artery bypass grafting; CAD: coronary artery disease; PCI: percutaneous coronary intervention.

Variable	Total N = 57 patients	60-79 years, n = 30 patients	≥80 years, n = 27 patients
Non-obstructive CAD/plaque	8 (14.0%)	3 (10.0%)	5 (18.5%)
Single-vessel disease	28 (49.1%)	19 (63.3%)	9 (33.3%)
Double-vessel disease	12 (21.1%)	1 (3.3%)	11 (40.7%)
Triple-vessel disease	9 (15.8%)	7 (23.3%)	2 (7.4%)
PCI	43 (75.4%)	24 (80.0%)	19 (70.4%)
CABG	2 (3.5%)	1 (3.3%)	1 (3.7%)
Optimal medical therapy	12 (21.1%)	5 (16.7%)	7 (25.9%)

## Discussion

The present prospective observational study compared the demographic, clinical, echocardiographic, and coronary angiographic characteristics of elderly (60-79 years) and very elderly (≥80 years) patients with CAD presenting to a tertiary care center in North India. The principal findings were that hypertension remained the most prevalent cardiovascular risk factor in both age groups; elderly patients more frequently exhibited single-vessel CAD, whereas very elderly patients demonstrated a higher proportion of double-vessel disease. Despite these differences in angiographic disease burden, left ventricular systolic function and regional wall motion abnormalities were broadly comparable between the two groups. In addition, dyspnea and angina were the predominant presenting symptoms, while typical chest pain was less common, highlighting the atypical clinical presentation of CAD in older adults.

The predominance of dyspnea (63.2%) and angina (50.9%), together with the relatively low frequency of typical chest pain (31.6%), is consistent with previous reports demonstrating that elderly patients frequently present with atypical manifestations of CAD. Chaudhary et al. [8] reported chest pain in 74% and dyspnea in 50% of patients aged >75 years presenting with acute coronary syndrome, while the French ONACI registry similarly demonstrated an increasing burden of CAD with advancing age. These findings suggest that clinicians should maintain a high index of suspicion for CAD in elderly individuals presenting with non-classical symptoms, thereby facilitating earlier diagnosis and timely management [9].

Although the present study did not specifically evaluate silent myocardial ischemia, the relatively low prevalence of typical chest pain and predominance of dyspnea support previous observations that CAD in very elderly patients frequently presents atypically. Age-related alterations in pain perception, autonomic dysfunction, multiple comorbidities, and the higher prevalence of diabetes mellitus may contribute to silent or less typical ischemic presentations in octogenarians. Consequently, clinicians should maintain a high index of suspicion when evaluating very elderly patients presenting with unexplained dyspnea, fatigue, or other nonspecific symptoms, as delayed recognition of myocardial ischemia may adversely affect clinical outcomes.

Hypertension was the most prevalent risk factor (75.4%), consistent with the INTERHEART South Asian cohort, and was marginally higher in the elderly group [10]. Bodkhe et al. reported hypertension in 86% of patients with CAD, while tobacco use was observed in 29% in their study population, which is comparable to our findings [11]. In another study by Ravi et al., hypertension was shown in 61%, diabetes in 43%, tobacco use in 36%, and family history of CAD in 20% of females with CAD [12]. In our study, dyslipidemia was relatively more common among the very elderly, whereas diabetes mellitus predominated in the elderly group. Diabetes remains an important contributor to accelerated coronary atherosclerosis and adverse cardiovascular outcomes because of its association with diffuse CAD and left ventricular dysfunction [13]. Smoking was rare and predominantly observed in the younger elderly group, possibly reflecting survivor bias and changing smoking patterns. Family history of CAD, although less frequent in the very elderly, remains an important consideration given the shared genetic and environmental influences on atherosclerotic risk.

Echocardiographic assessment demonstrated broadly comparable left ventricular systolic function and regional wall motion abnormalities between the elderly and very elderly groups. However, coronary angiography revealed differences in disease distribution. Single-vessel disease was more frequently observed among patients aged 60-79 years, whereas double-vessel disease predominated in patients aged ≥80 years, suggesting a greater burden of coronary atherosclerosis with advancing age. LAD was the most frequently affected vessel in both groups, consistent with the findings reported by Saghir et al., and with established pathophysiological susceptibility of LAD to atherosclerotic plaque formation in the South Asian population [14]. LAD (41%) was the most common vessel, followed by RCA (29%) [12]. LAD was the most commonly affected, as per the study by Pinto et al. as well [15].

Despite the higher coronary risk, a study on acute myocardial infarction in octogenarians by Matetzky et al. has shown that elderly patients benefit greatly from PCI in cases of acute myocardial infarction [16]. Our study demonstrated that 43.3% of elderly patients (60-79 years) and 55.6% of very elderly patients (≥80 years) presented with acute coronary syndrome, indicating a substantial burden in both groups. This finding highlights the need for timely diagnosis and intervention, as prompt management of acute coronary syndrome can significantly improve outcomes even in advanced age.

The greater burden of multi-vessel disease in the very elderly likely reflects the cumulative effect of prolonged exposure to cardiovascular risk factors and age-related vascular changes. Notably, the discordance between angiographic severity and echocardiographic dysfunction in the very elderly suggests potential compensatory myocardial mechanisms, as well as limitations in current functional assessment modalities in this age group.

The high prevalence of conventional cardiovascular risk factors across both elderly age groups emphasizes the continued importance of aggressive risk factor modification, guideline-directed medical therapy, and timely coronary evaluation, irrespective of age. The greater burden of multivessel CAD and the predominance of atypical clinical presentations among very elderly patients further highlight the need for individualized diagnostic and therapeutic strategies in this rapidly growing patient population. As life expectancy continues to increase, optimizing cardiovascular care in octogenarians will become increasingly important to improve both quality of life and clinical outcomes.

Limitations

This study has several limitations. First, it was conducted at a single tertiary care center with a relatively small sample size, which may limit the generalizability of the findings. Although the calculated sample size was 91 participants, only 57 eligible patients were enrolled during the study period, which may have reduced the statistical power of the study. Furthermore, comprehensive comparative statistical analyses, including multivariable adjustment, confidence interval estimation, and effect size analysis, were not performed. Therefore, the findings should be interpreted as descriptive observations rather than definitive evidence of age-related differences. In addition, patients with peripheral vascular disease, abdominal aortic aneurysm, transient ischemic attack, stroke, and chronic kidney disease were excluded to minimize potential confounding. Consequently, the study cohort represents a selected population and may not fully reflect the broader spectrum of elderly patients with CAD encountered in routine clinical practice. Therefore, the generalizability of the findings should be interpreted with appropriate caution. Finally, the observational design allows identification of associations but does not establish causal relationships. Larger multicenter studies with adequate statistical power are required to validate these findings.

## Conclusions

This prospective observational study provides descriptive insights into the clinical, echocardiographic, and coronary angiographic characteristics of elderly and very elderly patients with CAD. Elderly patients demonstrated a higher frequency of single-vessel disease, whereas very elderly patients more commonly exhibited double-vessel disease, while left ventricular systolic function remained broadly comparable between the groups. These findings should be interpreted within the context of the study's observational design, relatively small sample size, and descriptive analysis. Larger multicenter studies are warranted to validate these observations and further characterize CAD in the growing very elderly population.
